# Recent Advances in Catalyzed Sequential Reactions and the Potential Use of Tetrapyrrolic Macrocycles as Catalysts

**DOI:** 10.3390/molecules23112796

**Published:** 2018-10-28

**Authors:** Everton Henrique Santos, Charles Carvalho, Carolina Machado Terzi, Shirley Nakagaki

**Affiliations:** Laboratório de Bioinorgânica e Catálise, Departamento de Química, Centro Politécnico, Universidade Federal do Paraná (UFPR), Curitiba, Paraná 81531-990, Brazil; everhs1@gmail.com (E.H.S.); charlesscarvalhoo@gmail.com (C.C.); carolterzi97@gmail.com (C.M.T.)

**Keywords:** tetrapyrrolic macrocycles, porphyrin, catalysis, tandem, cascade reaction, oxidation

## Abstract

Complexes of porphyrins and of other similar tetrapyrrolic macrocycles are extensively explored as catalysts for different chemical processes, and the development of solid catalysts for heterogeneous processes using molecules with the ability to act as multifunctional catalysts in one-pot reactions is increasing and can lead to the wider use of this class of molecules as catalysts. This mini review focuses on the application of this class of complexes as catalysts in a variety of sequential one-pot reactions.

## 1. Introduction

Catalysis is applicable to all chemical processes and contributes enormously to sustainable chemistry, enabling different chemical transformations, and reducing the need to use stoichiometric reactions for the manufacture of fine chemicals and pharmaceuticals. This is in concordance with the ninth principle of green chemistry [1], and is considered to be one of the most important tools to implement it, since catalytic systems often require lower amounts of reactants and external energy, in addition to fewer steps, playing an important role in environmental sustainability [2,3,4].

Catalysts can be classified as homo- and heterogeneous and enzymatic catalysts [5]. The most explored type of reaction system is homogeneous, since it often exhibits higher yield and selectivity. In terms of scaling up research and development of products, however, homogeneous catalytic processes have a considerable disadvantage: the difficulties of recovering the catalyst for reuse, since it is in the same phase as the other reactants and products, making it an arduous process that is usually not cost-effective [6]. At the same time, in homogeneous catalysis, the catalytic sites in the catalyst species are frequently unprotected, enabling undesirable secondary reactions to happen, such as deactivation, poisoning, side reactions, dimerization, etc. [7]. In this context, many efforts were made by researchers to propose heterogeneous processes to overcome these problems. Heterogeneous catalytic systems facilitate catalyst recovery via simple processes, such as filtration [6], decantation [8], and magnetic recovery [9] (if the solid is a magnetic particle), among others.

There are many reports of the preparation of solid catalysts via their immobilization onto inert and usually inorganic matrices, such as layered double hydroxides [10], mesoporous silica [11,12], titanium oxides [8], zeolites [13], magnetic particles [14], and many more [15,16,17,18], in order to recover the catalyst for reuse. In addition, catalyst immobilization on inert matrices can produce unusual activities and synergism between the interacting species [19].

Regarding the chemical approaches investigated, most catalytic systems are still based on homo- and heterogeneous single-step reactions [6], and the recovery and purification of catalysts for reuse is still expensive and time-demanding, making them unsuitable for industrial use. Based on this, research is expanding on reactions that involve multiple steps. 

The main inspiration for the investigation of multifunctional processes is natural systems. In nature, there are many molecules that can act as catalysts in multistep reactions in mild conditions with high yields in particular enzymes. In enzyme systems, there are specific activated sites for specific reactions [20]. 

Taking the cytochrome P450 enzyme family as an example, they are present in all life forms and are responsible for catalyzing many reactions, such as the hydroxylation of saturated hydrogen carbon, epoxidation of double bonds, *O*-dealkylation, and *N*-dealkylation, among others [21,22,23,24,25].

Mimicking the structure and activity of such enzymes can be an important tool to propose synthetic molecules capable of multistep or sequential reactions, such as that which occurs in the generation of dienes via enzymatic oxidation. This reaction is initiated by the oxidation of phenols to generate ortho-quinones, which in turn undergo hydroxylation–oxidation combined with a Diels–Alder reaction to yield the desired bicyclic cycloaddition product [26].

In these natural systems, the reactions occur without separation of intermediates, which are simultaneously present in the same environment with other reactants. This kind of naturally occurring reaction in biological systems can be compared, in rough terms, to the chemical reactions classified as one-pot, presenting high catalyst selectivity and high yields in natural environments. 

Synthetic metalloporphyrins and other macrocycles (tetrapyrrolic or not) were described as excellent cytochrome P450 biomimetic catalysts to promote a selective and efficient family of reactions, such as olefin epoxidation and alkane (linear and cyclic) hydroxylation [27,28,29,30,31,32]. Despite the many reports, there are few examples of the design and preparation of efficient catalytic systems able to promote sequential one-pot reactions involving catalyst species based on metalloporphyrins. The challenge is bigger if the catalyst system is heterogeneous and recyclable.

In this mini review, the progress of this catalytic field is reviewed, focusing on some representative examples of the use of this versatile family of catalysts—macrocycle complexes based on pyrrole (Section 4). Before this, some definitions and terms necessary to understand tetrapyrrolic macrocycle systems are introduced (Section 2). The authors found it necessary to introduce some definitions because different concepts were found for similar sequential reactions [33]. In Section 3, some examples involving non-tetrapyrrolic catalytic systems are used for the purpose of clarifying the one-pot sequential catalytic process. 

## 2. Biological and Some Synthetic Tetrapyrrolic Macrocycle Systems

**Figure 1:**
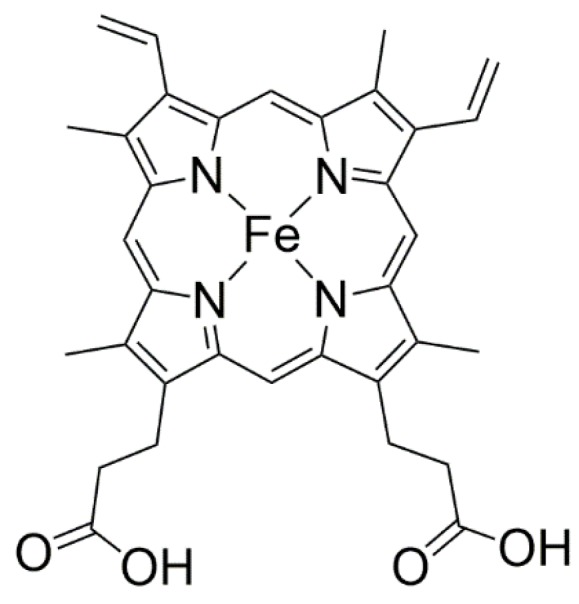
Schematic representation of iron(II) complex of protoporphyrin IX.

Since the 1960s, after the discovery of a hemeprotein able to catalyze the selective hydroxylation of substrates, such as drugs and steroid hormones [34], biological enzyme systems were explored as inspiration by chemists to develop catalytic system models, able to perform in vitro the same catalytic reactions observed in vivo, with high selectivity and efficiency under mild conditions. This hemeprotein was identified as being composed of a metalloenzyme based on the iron(II) complex of a porphyrin called protoporphyrin IX (Figure 1), which acts as a cofactor of a family of enzymes known as cytochrome P450.

The cytochrome P450 enzyme family plays a vital role in most living organisms, performing a huge diversity of biological functions, ranging from catalyzing reactions involved in biosynthesis metabolic pathways to the biodegradation of endogenous compounds and the metabolism of xenobiotic compounds [35,36,37]. 

The protoporphyrin IX complex is structurally composed of an aromatic macrocyclic ring consisting of four pyrrole units linked by four methinic bridges. This macrocycle, known as porphyrin, has a cavity whose diameter is about 4.0 Å, capable of accommodating more than 50 metal ions, leading to the formation of stable complexes named metalloporphyrins [35,38].

Efforts to build catalytic system models based on the reactivity of the cytochrome P450 enzyme family led to a profound knowledge of the mechanism and nature of these catalytic species. One of the first catalyst models able to mimic the cytochrome P450 catalytic activity was developed by Groves and coworkers in 1979, where they showed the catalytic activity of the synthetic iron(III) complex of *meso*-tetraphenylporphyrin ([Fe(TPP)]Cl), which was able to catalyze the hydroxylation and epoxidation of hydrocarbons with iodosylbenzene as an oxygen source under mild conditions [39].

**Figure 2:**
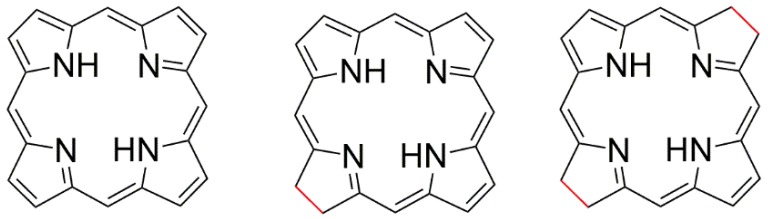
Structures of porphyrin, chlorin, and bacteriochlorin from left to right.

There are compounds with different variations in the structure of tetrapyrrolic macrocycles that give rise to distinct compounds and chemical behavior in vitro with similar structures in vivo. They are known as chlorin when one of the pyrrolic units is reduced, or bacteriochlorin when two of the pyrrolic rings at opposite positions are reduced, as illustrated in Figure 2 [40].

**Figure 3:**
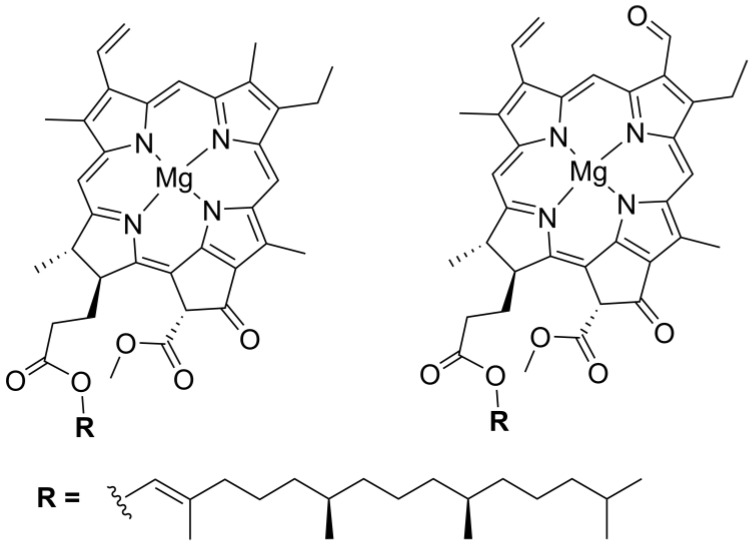
Schematic representation of the structures of chlorophyll a (**left**) and chlorophyll b (**right**).

In nature, substituted chlorin macrocycles are an important component of chlorophylls (Figure 3), where they form complexes via coordination with magnesium ions and participate in physiological functions of plants through photosynthesis, employing solar energy to convert carbon dioxide and water into carbohydrates and dioxygen as byproducts [40,41].

**Figure 4:**
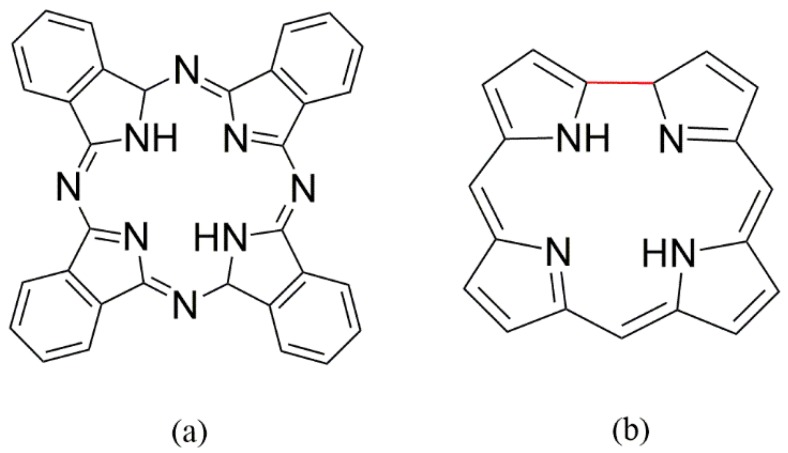
Structural representation of phthalocyanine (**a**) and a corrole (**b**).

The success of studies using synthetic metalloporphyrins as biomimetic catalysts based on cytochrome P450 systems prompted investigation of other macrocyclic systems as catalysts. In this way, other purely synthetic porphyrinoid complexes are being explored as potential catalysts of oxidation reactions [42]. For example, the macrocycle system named phthalocyanine exhibits structural analogy with porphyrin complexes. The different ring structures are obtained through the use of distinct reactants: pyrroles and aldehydes for porphyrins, and phthalic acids, phthalic anhydrides, or phthalonitriles for phthalocyanines (Figure 4a). The main advantage of this class of compounds is the synthetic method employed to obtain them, which allows good yields in just one reaction step. The procedure is based on a cyclotetramerization reaction among the precursors in the presence of a metal salt, which acts as a template for the macrocycle formation [42].

This class of compounds was already used to remove sulfur from petroleum fractions, by catalyzing the oxidation reaction of aromatic thiols, which are the main organic sulfur contaminant in petroleum derivatives [43].

Another porphyrinoid macrocycle intensively studied in recent years is the corrole family. This synthetic compound has structural features similar to porphyrin, but has one fewer methinic bridge in relation to porphyrin (Figure 4b) [44]. The common synthesis method for the preparation of *meso*-substituted corroles is based on a one-pot procedure involving two distinct steps. The first step involves a condensation reaction between the aldehyde and the pyrrole to produce oligo condensate tetrapyrranes. In the second step, oxidative ring closure occurs [45].

Like porphyrins, corroles can form complexes with a wide range of metallic ions, leading to the formation of metallocorroles [44]. Due to the structural similarity, the chemical properties of corroles and metallocorroles are also, in many aspects, similar to those of porphyrins and metalloporphyrins, making this class of compound useful in different fields of catalysis.

Nozaki et al. [46] demonstrated the use of manganese–corrole complexes acting as efficient catalysts in the polymerization reaction of epoxides, leading to the formation of polyethers, polycarbonates, and polyesters in the presence of a co-catalyst. Moreover, another study examined other properties of these compounds, such as for photodynamic therapy, where they act as sensitizers [47].

All these porphyrinoid-related macrocycles are known to have a particular spectral characteristic due to the high electronic conjugation of their structure. They possess strong absorption ranging from 300 nm to 800 nm in electromagnetic spectra, depending on the macrocycle structure. These particular patterns of absorption are frequently used to characterize these compounds. For instance, porphyrins and chlorins possess intense absorption in the region of 400–450 nm due a Soret band, as well as 500–700 nm originating from the Q bands. Other related macrocycles like phthalocyanines present the most intense absorption band between 550 and 800 nm [40]. Due to their intense absorption ranging in the ultraviolet-visible (UV-Vis) region, this class of compounds can be used as photosensitizers and can be applied in different fields, such as for photocatalysis [48] or photodynamic therapy [47,49,50].

## 3. One-Pot Sequential Reactions

The term “one pot” derives from reactions observed in biological systems in cases that involve a process in which all reactants, as well as catalysts, are present in the reaction medium from the outset. In these reactions, due to catalyst selectivity, both species do not interfere with each other, no matter how many mechanisms compose the process. In this sense, there is only one necessary step for isolation and purification of intermediates and catalysts, making it environmentally and economically attractive due to lower cost and time and the reduced need for reactants [4,33,51,52,53].

**Figure 5:**
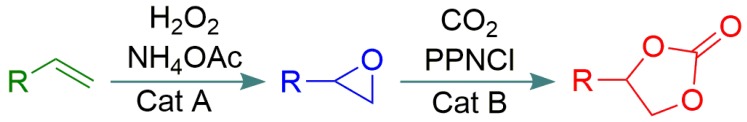
Schematic representation of a sequential reaction involving two catalytic species based on metalloporphyrins, one of manganese (Cat A) and the other of chromium (Cat B), where PPNCl is [(Ph_3_P)_2_N]Cl [54].

In contrast to one-pot sequential reactions, there are sequential reactions that are out of the one-pot scope, such as the reaction described by Dias and coworkers in which they synthetized porphyrin-based magnetic nanocomposites to be applied in sequential olefin epoxidation, followed by CO_2_ cycloaddition to epoxides, leading to the desired cyclic carbonates, as shown in Figure 5. Between the two steps, the solvent was evaporated and the mixture was transferred to another recipient containing a second catalyst to continue the next step. This kind of reaction, for example, leads to 70% yield [54].

In the one-pot context, sequential reactions encompass catalytic reactions with single or multiple mechanisms for all steps, using one (multifunctional or not) or more than one catalyst species, but, as said before, all from the outset.

In the context of one-pot reactions, there are different kinds of sequential reactions based on the possibility of single or multiple mechanisms with one or more catalysts and catalytic cycles. Hence, there are two major kinds of one-pot sequential reactions: domino (or the closely related cascade) and tandem reactions [33,55,56].

Those involving one kind of mechanism and one catalyst are usually called domino or cascade [1,33,57], in which there is no extra reactant addition during the reaction.

The term “domino” is also subdivided into two different kinds of reactions: intra- and intermolecular. The first one designates reactions where the substrate undergoes multiple molecular transformations caused by a single catalyst, giving birth to various (n) transition states that lead to a product. The second one designates reactions involving multiples steps with one catalytic mechanism acting repeatedly, generating intermediates that are stable and can be isolated (in intermolecular reactions, it is also possible for molecules to undergo multiple transformations).

**Figure 6:**
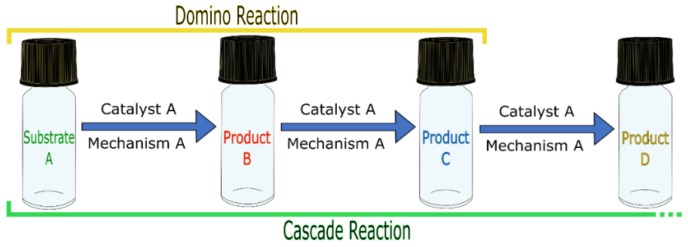
Schematic representation of domino and cascade catalyzed reactions.

In this sense, the term domino reaction is applied to reactions where the mechanism triggered by a single catalyst action repeats twice, as shown in Figure 6.

Following the same logic, the term “cascade” is almost synonymous with domino; however, it is reserved for reactions containing three or more single catalytic mechanism steps [58,59,60], such as that schematized in Figure 6.

**Figure 7:**
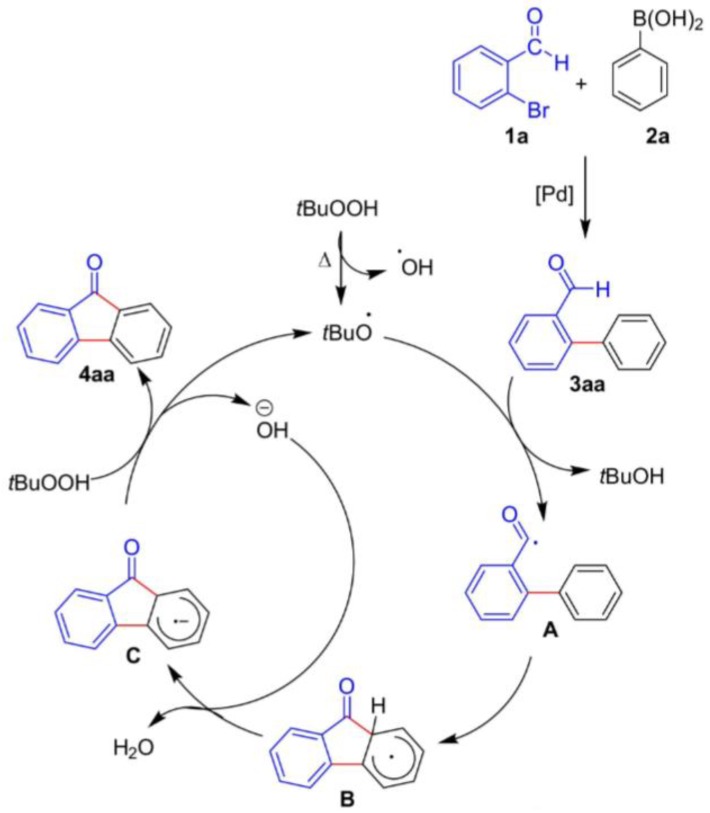
Catalytic cycle to obtain fluorenones from 2-bromobenzaldehyde (1a) and phenylboronic acid (2a) using palladium as a catalyst and *tert*-butyl hydroperoxide as an oxidant, following the domino one-pot concept (Kumar and Satyanarayana; Palladium Catalysis: One-Pot Synthesis of Fluorenones. Chemistry Select. 2018.3.7867-7870. Copyright Wiley-VCH Verlag GmbH & Co. KGaA. Reproduced with permission) [61].

To exemplify the domino reaction (which can be extend to the cascade reaction), in 2018, Kumar and Satyanarayana [61] reported the obtainment of fluorenones, which have meaningful interest in the biomedical and material fields, from 2-bromobenzaldehyde (Figure 7, 1a) and phenylboronic acid (Figure 7, 2a), using palladium as a catalyst and *tert*-butyl hydroperoxide as an oxidant. Following the domino one-pot concept, after the catalyst’s action to form the Suzuki product (Figure 7, 3aa), the molecules undergo intramolecular transformations (Figure 7, steps A–C) to generate the desired product (Figure 7, 4aa), with 86% yield in the best condition studied. The authors also proposed the mechanism represented in Figure 7 [61].

On the other hand, reactions in which two or more distinct mechanisms act on the substrate are called tandem reactions. They are subdivided into three classes: orthogonal tandem, auto tandem, and assisted tandem [4,33,51,55,56].

**Figure 8:**
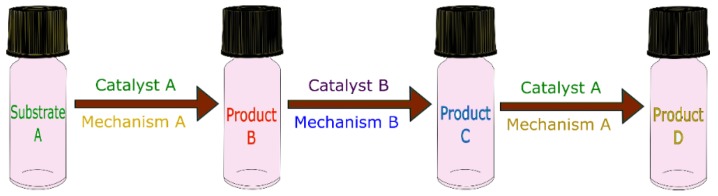
Schematic representation of an orthogonal tandem catalytic reaction.

Orthogonal tandem refers to reactions with multiple catalysts working together with mutual independence, in which one catalyst does not react before a specific product is formed. In other words, it also can be seen as “one after another” (Figure 8) [33,55].

**Figure 9:**
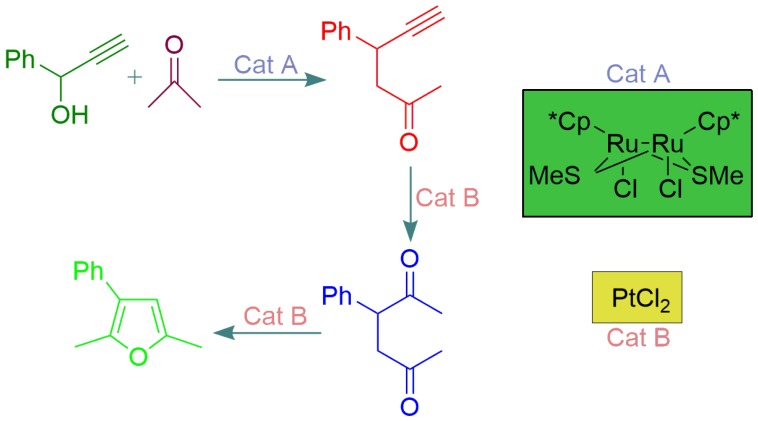
Schematic representation of the orthogonal tandem catalytic reaction using two different catalysts, Cat A (ruthenium complex) and Cat B (Pt compound), to obtain furan compounds. The scheme is based on Reference [62], where Cp = η_5_-C_5_Me_5_ and MeS = CH_3_S.

As an example of an orthogonal tandem catalytic reaction, in 2003, Nishibayashi et al. [62] reported the reaction between 1-phenyl-2-propyn-1-ol and a ketone to obtain a specific furan, depending on the ketone used. In this process, they used two catalysts, an Ru complex and PtCl_2_ (Figure 9). Both catalysts were simultaneously present in the reaction medium, and the Pt catalyst acted only after the formation of an intermediate species via catalytic action of the Ru complex [62].

**Figure 10:**
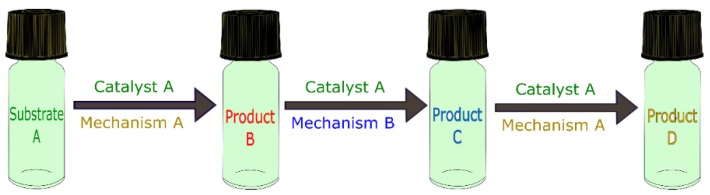
Schematic representation of the auto tandem catalytic reaction.

Auto tandem is a term reserved for reactions with a single multifunctional catalyst. This catalyst has two or more activated catalytic sites in its structure; thus, it can cause different catalytic reactions in the substrate, as exemplified in Figure 10 [33].

**Figure 11:**
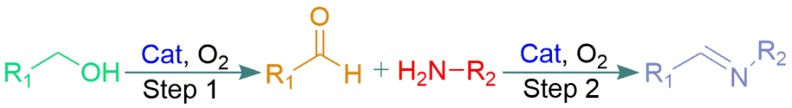
Schematic representation of the auto tandem catalytic reaction using an Au compound as catalyst (Cat = Au/zinc–aluminum hydrotalcite (HT)) to obtain aldehyde–amine condensation products [63].

Liu et al. [63] demonstrated the application of a multifunctional catalyst prepared with gold nanoparticles immobilized on zinc–aluminum hydrotalcite (Au/HT) in an auto tandem reaction for the preparation of aldehyde–amine condensation products. The catalytic reaction comprised two steps: the first occurred via proton abstraction from benzyl alcohol (R_1_C-OH, Figure 11), forming aldehyde, promoted by basic catalyst sites in hydrotalcite (Au/HT = Cat), to react with 1-hexylamine (H_2_N-R_2_); the second depended on the gold nanoparticles (Cat) acting as Lewis acid sites to activate the N-H bond in the amine to condense it with the formed aldehyde, leading to the desired condensation product (imine), as summarized in the mechanism depicted in Figure 11 [63]. 

**Figure 12:**
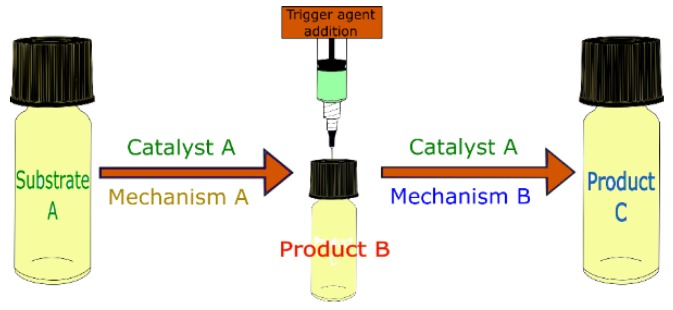
Schematic representation of an assisted tandem catalytic reaction.

When there is a need to add a reactant to trigger the activity of a second catalytic site in the same catalyst or to change the catalyst to propitiate a second catalytic function, starting a second reaction step, the process is called assisted tandem. This kind of reaction only involves the use of a single multifunctional catalyst, as illustrated in Figure 12 [33].

**Figure 13:**
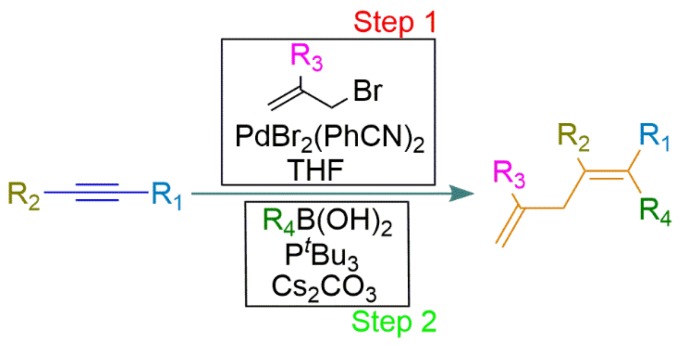
Schematic representation of the assisted tandem catalytic reaction using a Pd compound as a catalyst and *tert*-butylphosphine (PtBu_3_) as a trigger reactant to obtain tetra-substituted alkenes, where R_1_ to R_4_ are different substituents [64].

It is important to highlight that the second stage of catalytic activity does not occur before addition of the triggering agent. To contextualize this, in 2002, Thadani and Rawal reported an assisted tandem reaction associated with bromo- or chloroallylation followed by Suzuki cross-coupling reaction to obtain tetra-substituted alkenes (Figure 13). In the first step, Pd^II^ was applied as catalyst and, after its conclusion, in the same flask, *tert*-butylphosphine was added to reduce the Pd^II^ to Pd^0^, used as the catalytic species for the following step [64].

## 4. Uses of Tetrapyrrolic Macrocycles in Sequential Reactions

**Figure 14:**
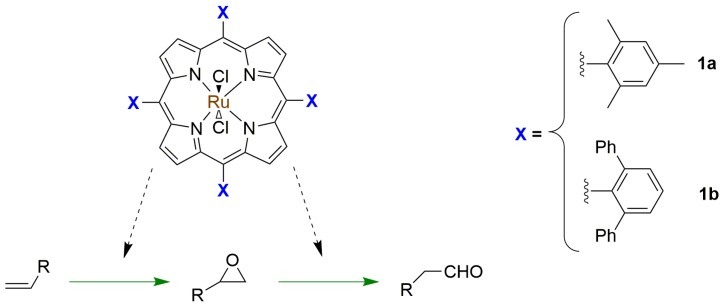
Schematic representation of a tandem reaction catalyzed by an Ru-porphyrin, adapted from Reference [65].

In 2008, Jiang and coworkers reported the use of a ruthenium porphyrin applied to the homogeneous aerobic-oxidation catalysis of terminal alkenes to aldehydes in a tandem epoxidation–isomerization approach [65]. The objective was to create an easier approach to achieve selectivity for aldehydes in metal-catalyzed oxidation reactions of 1-alkenes that use dioxygen as the sole terminal oxidant under mild conditions, since the process commonly also produces methyl ketones instead of only aldehydes (Figure 14). The authors studied different oxidation reaction conditions with ruthenium porphyrins, changing the solvent and the additives, and observed better yields using [Ru^IV^(TMP)Cl_2_] (TMP = tetramesithylporphyrin, Figure 14, 1a) as a catalyst in CDCl_3_ with addition of aqueous NaHCO_3_. A closer examination of that reaction over time using ^1^H-NMR spectroscopy revealed that, in the presence of NaHCO_3_, [Ru^IV^(TMP)Cl_2_] was oxidized to [Ru^IV^(TMP)O_2_], which led to the tandem epoxidation–isomerization reaction. The species generated in situ was responsible for the epoxidation step; however, the active intermediate for the epoxide isomerization step was unclear. They also concluded that the catalyst [Ru^IV^(TMP)O_2_] presented better reaction yield when generated in situ. Furthermore, they developed a new, recyclable ruthenium porphyrin catalyst (based on porphyrin 1b, Figure 14), [Ru^IV^(TMTTP)O,], which followed the same tandem pathway and whose reaction yields were above 90%, even after being recycled five times. In conclusion, that work is an example of how assisted tandem reactions with porphyrins as catalysts can be useful to achieve the desired selectivity. The authors successfully synthesized a new efficient and recyclable catalyst, used in one of the first reported sequential tandem reactions based on a porphyrin macrocycle.

**Figure 15:**
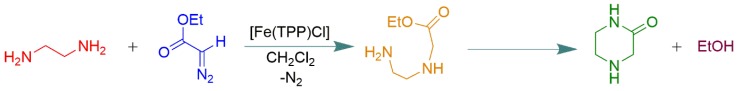
Schematic representation of a tandem insertion/cyclization reaction [66].

In 2010, Mbuvi and coworkers verified the potential of [Fe(TPP)Cl] as a catalyst in a tandem reaction involving N-H insertion in carbenes from diazo esters and its further cyclization, reporting a new one-pot approach for synthesizing heterocyclic compounds via the reaction of amines and the diazo reagents (Figure 15) [66]. As expected, the results showed that, using ethylenediamine and ethyl diazoacetate as the carbene source, the [Fe(TPP)Cl] acted as a highly efficient catalyst for the syntheses of 2-piperazinone, with yields above 80%. Since these products are important for their therapeutic properties, that study is a good example of how a known synthesis can be improved using a porphyrin catalyst in a one-pot tandem reaction. The authors demonstrated that the tandem N-H insertion/cyclization process is an easy one-pot procedure for the syntheses of novel piperazinones and their substituted analogs. Other examples can be found in a recent review concerning carbene transfer reactions catalyzed by dyes of the metalloporphyrin group [67]. 

**Figure 16:**
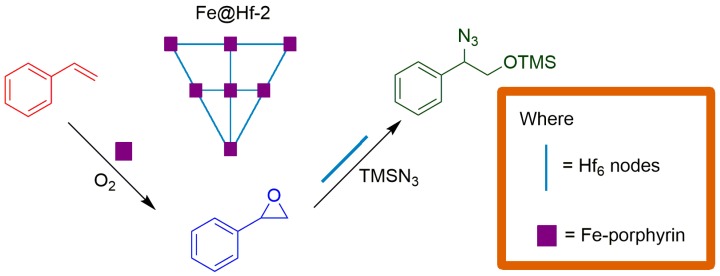
Schematic representation of a tandem reaction (olefin oxidation, di-alcohol formation, and protection with TMSN_3_) using the catalytic metal organic framework (MOF) solid Fe@Hf-2 [68].

In 2015, Beyzavi and coworkers reported the preparation and characterization of a robust metal organic framework (MOF) consisting of Fe^III^ porphyrin, and an Hf_6_ node decorated with iron ions. To synthetize it, they used [Fe(TCPP)Cl] (TCPP = 5,10,15,20-tetrakis(4-carboxyphenyl)porphyrin), HfOCl_2_∙8H_2_O, benzoic acid, *N*,*N*-diethylformamide, and iron ions, resulting in a solid with two additional Fe atoms coordinated to the Hf_6_ node of the framework, to give the overall formula C_96_H_64_Cl_4_Fe_4_Hf_6_N_8_O_32_ [68]. The resulting solid MOF, named Fe@Hf-2, was applied as a catalyst in a tandem reaction comprising styrene oxidation by Fe(III)porphyrin, followed by epoxide ring opening by Hf nodes/iron ions of the structured MOF, followed by the di-alcohol protection reaction using the reactant TMSN_3_ (azidotrimethylsilane), resulting in the protected azidohydrin product, which is an important precursor to α-amino alcohols (Figure 16). The Fe@Hf-2 solid showed catalytic activity in the epoxidation reaction with regioselectivity. This work is an example of the application of iron porphyrins in sequential reactions, as depicted by the orthogonal tandem catalysis in Figure 8, where the first step is catalyzed by Fe(III) porphyrin, and, after the first product’s formation, Hf nodes/iron ions are responsible for the second catalytic step, yielding the protected alcohol product using TMSN_3_. The authors successfully demonstrated the applicability of tandem reactions and also the reusability of heterogeneous catalysts from styrene epoxidation utilizing molecular oxygen as a terminal oxidant and epoxide ring opening. 

**Figure 17:**
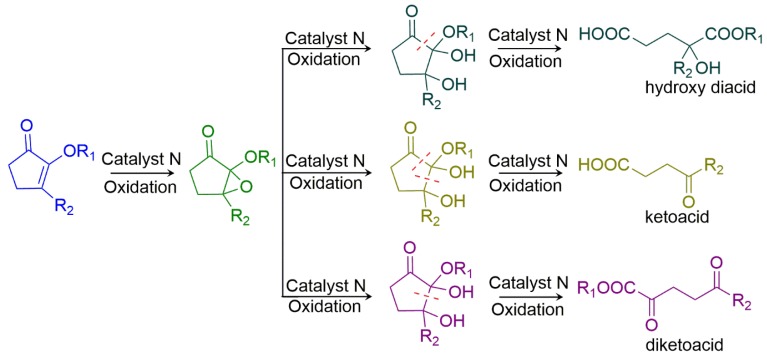
Schematic representation of the cascade catalytic reaction using Mn, Fe, or Co metalloporphyrin as a catalyst (catalyst N) in aerobic conditions to obtain the respective products, where R_1_ = H and R_2_ = Bn, Ph, Me, CH_2_CH_2_OBn, CH_2_CH_2_OH, CH_2_CH_2_NHBoc, CH_2_COOtBu, Cy, or Et. [59].

In 2018, Maljutenko and coworkers reported the use of metalloporphyrins as catalysts in aerobic cascade oxidation of cyclopentane-1,2-dione at room temperature, for 24 h at normal air pressure, to produce the oxidation products: hydroxy diacids, ketoacids, and diketoacids (Figure 17) [59]. To accomplish this, they studied two different porphyrin structures, octaethylporphyrin (OEP) and *meso*-tetraphenylporphyrin (TPP), metalated with Mn, Fe, and Co, generating six distinct catalysts. The Mn complexes showed at most 3% unreacted substrates, while Fe and Co catalysts showed between 20 and 49%. The best catalyst to obtain ketoacids was [Mn(OEP)Cl] (product yield 67%) using 5 mol% of catalyst. They also observed that [Co(TPP)Cl] was the most selective catalyst in diketoacid generation with 48% conversion (ketoacid: 24%; diacid: 8%). Even with 1 mol% of [Mn(TPP)Cl], they observed 55% hydroxy diacid conversion, but with low selectivity (ketoacid: 42%; diketoacid: 0%). Under the same experimental conditions, the non-catalyzed reaction presented only 19% yield with no selectivity in aerobic oxidation. In an effort to improve the selectivity to the desired hydroxy diacid product, they investigated the best solvent to use in the reaction. The use of [Mn(TPP)Cl] 1 mol% in toluene at room temperature improved the selectivity to hydroxy acid, with yield up to 76%. In this context, the authors reported an environmentally friendly cascade aerobic reaction involving metalloporphyrin in homogeneous catalysis. This work is interesting since the authors proposed that metalloporphyrins (in this case, the best one was [Mn(TPP)Cl]) can promote the cascade oxidation of cyclopentane-1,2-diones to obtain desired products (Figure 17), similar to the cascade reaction shown in Figure 6.

**Figure 18:**
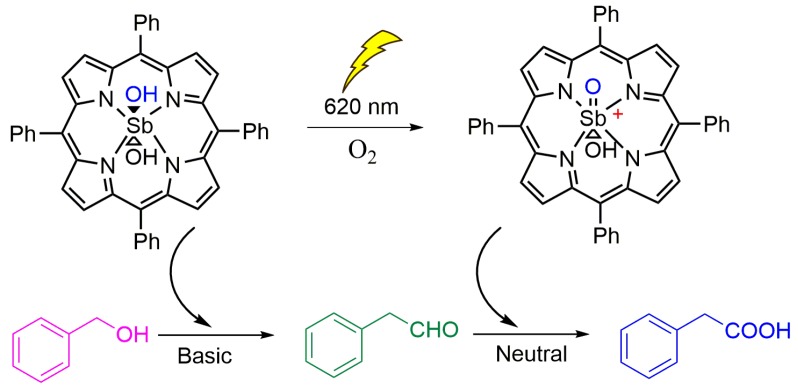
Schematic representation of a cascade reaction assisted by light irradiation. The first step occurs in basic pH, and the second one occurs in neutral pH [69].

In 2012, Hajimohammadi and colleagues described the use of Sb porphyrin as catalyst in a bio-inspired homogeneous pH-dependent cascade photoredox reaction to oxidize alcohol to aldehyde and then to carboxylic acid, as represented in Figure 18 [69]. They used [Sb(TPP)(OH)_2_]^+^ in a solvent mixture of water–acetonitrile with the substrate benzyl alcohol, in the presence of O_2_. The catalytic mixture was irradiated with visible light (<620 nm) or sunlight and NaOH was used to deprotonate the Sb porphyrin to generate the photo-oxidant species [SbO(TPP)(OH)], in turn producing benzaldehyde from alcohol substrate as the first step. In the following step, benzaldehyde was oxidized to form benzoic acid, the final product of the cascade reaction, under the same conditions used in the first reaction, except for the solution’s cascade pH value, which was kept neutral. After 72 h of photocatalytic reaction, conversion of 92% was observed. The use of porphyrin as a photocatalyst in a cascade reaction can overcome many environmental problems, in addition to showing high turnover frequency and number of catalysts. The use of the mentioned porphyrin in a 72-h cascade oxidation photocatalytic reaction was successfully executed with high yields.

**Figure 19:**
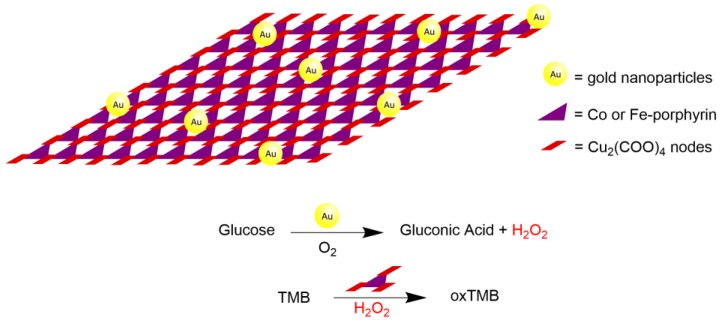
Schematic representation of two-dimensional (2D) nanosheet compound of Cu-TCPP(M) and gold nanoparticles applied as a catalyst in a sequential reaction [60].

Another interesting example of a catalytic cascade reaction was presented by Huang and coworkers recently. The authors reported the preparation of a new MOF solid based on metalloporphyrins and metal nanoparticles, such as gold, and their use in cascade reactions [60]. The authors found that the metalloporphyrinic part of MOF nanosheets can act as a peroxidase mimic, and Au nanoparticles can serve as artificial glucose oxidase, mimicking natural enzymes. The MOF was prepared via the incorporation of gold nanoparticles into metalloporphyrin two-dimensional (2D) MOF nanosheets previously prepared using [M(TCPP)] (M = Fe(III) or Co(III)) and Cu_2_(COO)_4_ paddlewheel clusters as metal nodes. The resulting 2D MOF-based hybrid nanomaterial, called Au NPs/Cu-TCPP(Fe), was applied in a 30-min cascade reaction, in which the first step was oxidation of glucose using O_2_ catalyzed by gold nanoparticles, mimicking glucose oxidase enzyme activity, generating gluconic acid and hydrogen peroxide. Then, hydrogen peroxide was used as an oxidant in the second step of the cascade to convert 3,3′,5,5′-tetramethylbenzidine (TMB) into its oxidized form, catalyzed by the Cu-TCPP(M) (Figure 19). The progress of the so-called artificial enzymatic cascade reaction was monitored using UV-vis spectroscopy to detect the final oxidation product. The authors reported that the MOF catalyst successfully catalyzed an enzymatic cascade reaction in mild conditions in a biomimetic system without any natural enzymes. It is also interesting to mention that the properties of each catalyst composing the MOF remained unchanged in the final MOF structure, which is important for possible reuse of this kind of catalyst. Although the authors reported this as a cascade reaction, it uses a multifunctional catalyst; therefore, we suggest it should be classified as auto tandem, based on the definitions regarding sequential reactions reported herein. 

**Figure 20:**
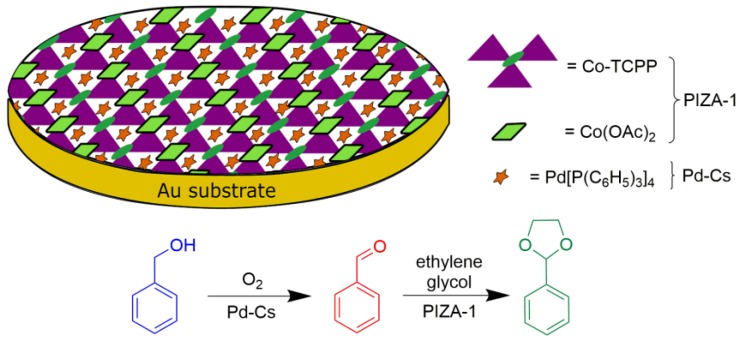
Schematic representation of Pd-Cs@PIZA-1 (Pd-Cs: Pd[P(C_6_H_5_)_3_]_4_; PIZA-1: porphyrinic Illinois zeolite analog) catalyst in an auto tandem where the first step is an oxidation followed by acetalization (Co-TCPP = cobalt(III)porphyrin) [70].

In a study involving the preparation of a tandem catalytic system, Vohra and coworkers developed a palladium catalyst encapsulated in a porphyrin network with a porphyrinic Illinois zeolite analog (PIZA-1) framework [70]. It was applied in a one-pot tandem oxidation–acetalization catalysis reaction (Figure 20). The three-dimensional (3D) structure of PIZA-1 was chosen because the authors envisioned the use of the solid microporous network as a host to the functional guest. The tetrakis(triphenylphosphine)-palladium, Pd[P(C_6_H_5_)_3_]_4_, abbreviated by the authors as Pd-Cs, was chosen as the guest for encapsulating the solid pores. The PIZA-1 solid, which was first reported by the Suslick group [71], was synthesized from cobalt(II) acetate and the free base porphyrin [H_2_(TCPP)], resulting in a solid containing Co^III^ porphyrin and trinuclear Co^II^-oxo clusters (Co-TCPP, Figure 20). To synthetize a thin film of the catalyst host-guest Pd-Cs@PIZA-1, the substrate was sprayed with cobalt(II) acetate, [H_2_(TCPP)] and Pd-Cs solutions to form a film using the liquid-phase epitaxial (LPE) encapsulation method in a modified epitaxial layer-by-layer encapsulation approach. The bifunctional capacity of the prepared material was studied to ascertain the catalytic activity of the guest Pd-Cs compound in the oxidation reaction of benzyl alcohol to benzaldehyde under oxygen atmosphere at 85 °C, followed by the Lewis acid catalysis of the host species PIZA-1, in the acetalization reaction of the resulting aldehyde with ethylene glycol. The authors observed conversion values of the acetalization product of 85%. In comparison, using only the PIZA-1 thin film without the guest species yielded no benzaldehyde, but showed 93% acetal product conversion when only the acetalization reaction was investigated. On the other hand, Pd-Cs species showed 100% alcohol conversion to aldehyde. The reusability of the Pd-Cs@PIZA-1 thin film catalyst was also examined, and showed only a slight catalytic activity reduction after the fourth cycle. That study is an interesting example of a sequential reaction using macrocycle compounds organized in a thin film. 

**Figure 21:**
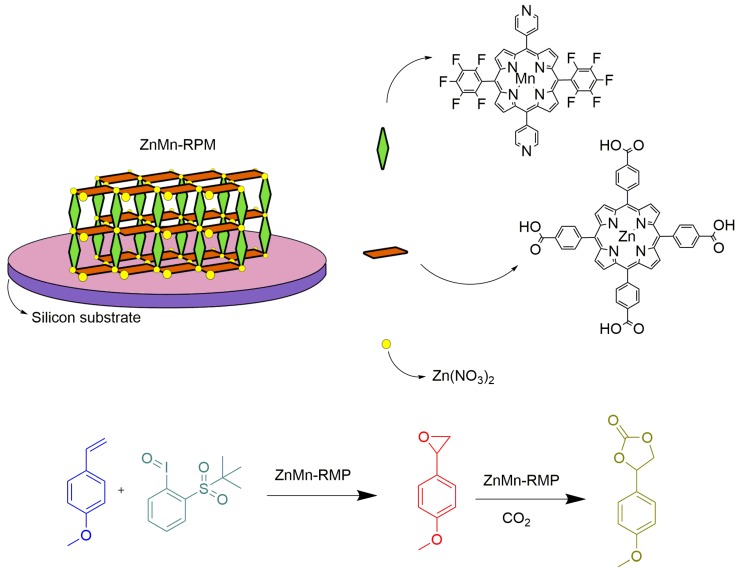
Schematic representation of MOF ZnMn-RPM (robust porphyrinic material) employed as a catalyst in a sequential reaction. [72].

Another interesting film material used as a catalyst in a sequential reaction was reported by Beyzavi and coworkers, combining the catalytic potential of two different metalloporphyrins in the same solid catalyst [72]. They prepared MOF material that was used for orthogonal tandem catalysis involving a manganese porphyrin to catalyze the epoxidation of an olefin substrate and a zinc porphyrin to catalyze epoxide ring opening and CO_2_ insertion. The MOF was composed of robust porphyrinic material (RPM-MOF), synthesized with [Zn(TCPP)], (Figure 21, red rectangles) and 5,15-dipyridyl-10,20-bis(pentafluorophenyl)porphyrinato mangenese(III) chloride (Figure 21, green lozenges) as the pillars, and zinc nitrate hexahydrate as nodes, receiving the name ZnMn-RPM, as shown in Figure 21. The material was prepared via two methods: solvothermal, resulting in a bulk material, and layer-by-layer, resulting in an ultrathin film on a self-assembled-monolayer-coated silicon platform. The authors compared the catalytic activity of the MOF material prepared as a bulk and as a film in the tandem reaction at 65 °C and 60 atm of CO_2_. Using *p*-methoxystyrene as the substrate and 1-(*tert*-butylsulfonyl)-2-iodosylbenzene as the terminal oxidant, they obtained the bulk solid as catalyst 4-(4-methoxyphenyl)-1,3-dioxolan-2-one as the desired carbonate product with more than 60% yield. On the other hand, using the film as catalyst they observed a turnover number higher than 4000, 40 times greater than the first catalytic solid. They concluded that the bulk solid with uncontrolled particle size and orientation caused a significant impact on the kinetics of the desired sequential reaction process, and the ultrathin film was more efficient in terms of diffusive accessibility of reactants to catalyst active sites, explaining the outstanding turnover number. Although they did not mention any recycle experiments, the catalytic system described is an interesting example of true tandem catalysis without the need to isolate intermediates or adjust reaction conditions. However, the tandem reported here is an auto tandem, since the different catalytic species are present in a single compound denominated as ZnMn-RPM, and not as isolated forms.

**Figure 22:**
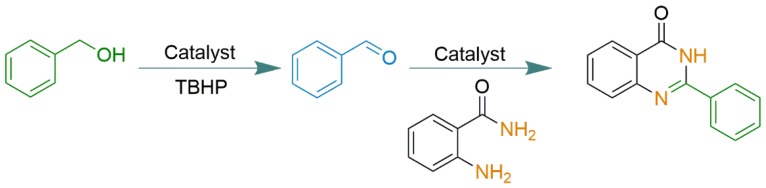
Schematic representation of a tandem process promoted by the porous organic polymer (POP) solid based on ironporphyrin, where iron acted as a redox and Lewis acid catalyst [73].

Oveisi and coworkers reported the synthesis of an Fe-porphyrin-based porous organic polymer (POP material), and used it as a catalyst in a tandem process involving an in situ oxidation/cyclic aminal formation/oxidation sequence, to convert benzyl alcohol to 2-phenyl-quinazolin-4(3*H*)-one [73]. They reported the preparation of a free base porphyrin POP material (Fb-PPOP) via a nucleophilic substitution reaction between tetrakis(pentafluorophenyl)porphyrin and hexahydroxytriphenylene. The resulting solid was metalated with iron(III), and the resulting polymeric solid was purified and characterized (named Fe-PPOP). The presence or absence of residual iron in the polymeric structure or the presence of unmetalated free base porphyrin in the material was not evaluated. The resulting POP material (Fe-PPOP) was used as a Lewis acid catalyst for methanolysis of ring opening of styrene oxide to convert it to β-methoxyalcohol. Since no Fe leaching was observed, such that the catalytic system was consistent with a heterogeneous process, the authors also reported that the Fe-PPOP solid could be reused without a significant decrease in its catalytic activity. The POP solid was also used for a tandem catalytic process in an oxidation/cyclic aminal formation/oxidation sequence (Figure 22). In this tandem example, the primary substrate benzyl alcohol was catalytically oxidized to ketone using *tert*-butyl hydroperoxide as an oxidant (TBHP). In the first step of the tandem process, the catalyst Fe-PPOP acted as a catalyst for the oxidation reaction, an activity commonly presented by iron porphyrins. In the second step of the catalytic reaction and in the presence of the second reactant *o*-aminobenzamide, the ketone reacted to produce 2-phenyl-quinazolin-4(3*H*)-one, with the solid Fe-PPOP acting as a Lewis acid catalyst, where both activities were performed by the iron site. The authors successfully synthesized a new type of multifunctional porphyrin-based catalyst, which could be recovered and reused without losing its catalytic activity.

**Figure 23:**
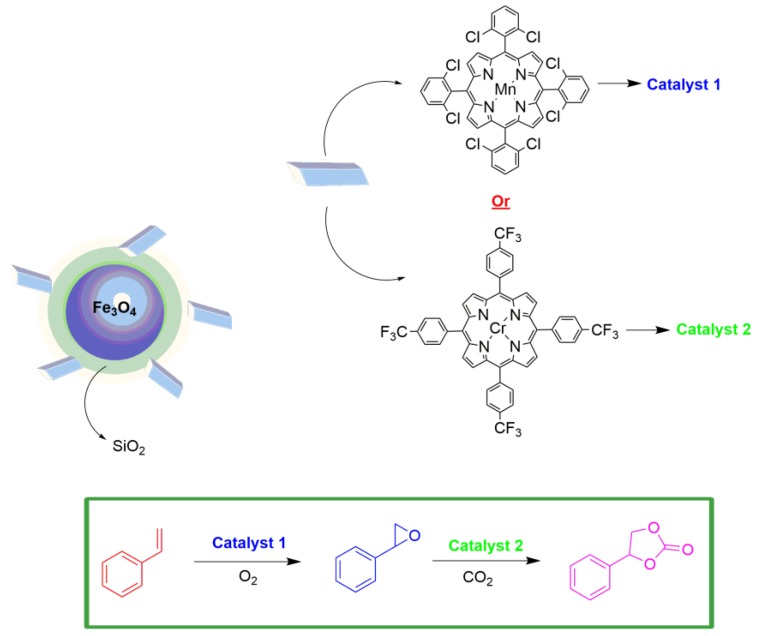
Schematic representation of MNP@SiO_2_-(M) and its application in a sequential reaction of epoxidation followed by CO_2_ insertion [54].

Recently, Dias et al. [54] reported a heterobimetallic catalytic system based on the combination of two hybrid materials, one resulting from the covalent immobilization of a manganese porphyrin on silica-modified magnetite, and the other via the immobilization of a chromium porphyrin (hybrid materials named MNP@SiO_2_-8Mn and MNP@SiO_2_-4Cr, respectively) (Figure 23). In the context of the transformation of carbon dioxide into organic chemicals to minimize its environmental impact, the authors proposed the preparation of cyclic carbonates, a valuable chemical with a range of applications (intermediates for fine chemicals, biomedical applications, uses as electrolytes for lithium batteries, fuel additives, green solvents, etc.) via the catalytic insertion of CO_2_ catalyzed by chromium porphyrin in epoxides obtained from the sequential olefin catalytic oxidation by manganese porphyrin. The solids catalysts were used to promote sequential transformation of styrene to epoxide using green oxidants such as O_2_ or H_2_O_2_ followed by the CO_2_ cycloaddition reaction, resulting in the cyclic carbonate. The authors stated there were no reports of the application of heterogeneous metalloporphyrin-based recyclable catalysts to promote the sequential transformation of olefins into cyclic carbonates, using green oxidants such as O_2_ or H_2_O_2_, as investigated in their work. They proved the recyclability of the solids in three consecutive cycles without significant loss of activity or selectivity. In this context, this work shows the usage of metalloporphyrin-based catalytic solids in a sequential catalytic reaction with good yield and reuse capacity. It is necessary to mention here that this process is a sequential reaction; however, as we already mentioned, it is out of the one-pot scope. 

**Figure 24:**
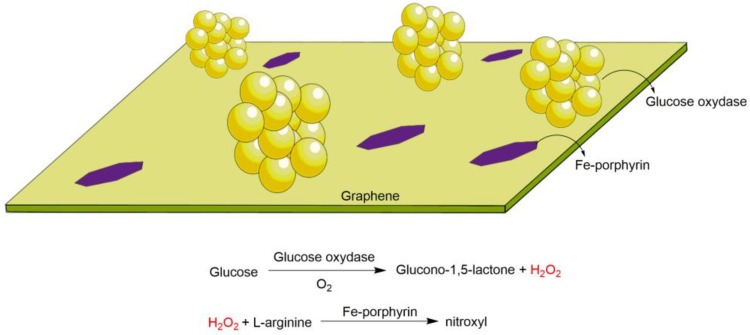
Schematic representation of solid catalytic graphene as a support for the Fe porphyrin and glucose oxidase. The latter one was employed in a glucose oxidation reaction, followed by Fe porphyrin acting on l-arginine oxidation to a nitroxyl species in the presence of H_2_O_2_ formed in the first step [74].

To finish the review of some examples of the use of macrocycle compounds as catalysts for sequential one-pot reactions, it is important to describe an elegant example based on enzyme/iron porphyrin/graphene working together to produce HNO (nitroxyl species), a relevant antithrombotic species analogous to NO, in an auto tandem catalytic system. In 2014, Xue and colleagues [74] reported the preparation, characterization, and sequential catalytic use of a solid resulting from the immobilization of glucose oxidase and hemin (iron(III) protoporphyrin IX) on graphene (Figure 24). The oxidation of l-arginine using hydrogen peroxide catalyzed by iron porphyrin was possible and resulted in HNO. The sequential reaction was confirmed since the hydrogen peroxide was produced in situ via the enzymatic activity of glucose oxidase acting in the presence of molecular oxygen to transform glucose into glucono-1,5-lactone (and H_2_O_2_). With this idea, the authors could conceive a system to produce HNO. Based on the bench results, they also expected that the catalyst could generate HNO using physiological glucose, l-arginine, and blood oxygen. For this purpose, they proposed embedding the catalyst in biocompatible films (polyurethane) to create a surface coating, offering a potential solution for the sustained generation of antithrombotic HNO species on medical devices when in contact with fresh blood. 

## 5. Final Remarks

In this mini review, some recent works employing metalloporphyrin macrocycles as catalysts in one-pot sequential reactions were presented. To support the analyses of the works, the definitions involved in the class of sequential one-pot reactions were reviewed.

From this review, it is possible to infer that, in recent years, although porphyrin complexes are versatile candidates for the creation of multifunctional catalysts for homogeneous or heterogeneous catalysis, few examples using these molecules were presented. Nevertheless, some interesting reports of one-pot sequential catalytic reactions were published for catalytic systems with different purposes, such as the preparation of desirable organic products, oxidation reactions using dioxygen as terminal oxidant, photoredox reactions, oxidation-isomerization, epoxidation-ring opening, oxidation-acetalization, N-H insertion into carbenes to prepare compounds with therapeutic properties, oxidation of 1,2-diones to produce diacids, ketoacids, and diketoacids, the production of HNO, an antithrombotic species analogous to NO, via l-arginine oxidation for the preparation of medical devices, etc. However, as presented in the different examples in Section 3, the potential of sequential catalysis can increase using porphyrins and metalloporphyrins and other tetrapyrrolic macrocycles, as is already the case for other catalytic species. 

Finally, more thorough research will certainly be carried out in the near future for the formulation of catalytic systems, mainly based on solid catalysts, resulting from the immobilization of one or more different metalloporphyrins, enzymes, or metalloporphyrins heterogenized in insoluble solids such as MOFs, polymers of intrinsic microporosity (PIMs), and POPs. In the future, these synthetic porphyrins and maybe other tetrapyrrolic macrocycles can act as multifunctional catalysts for a multipurpose catalytic process occurring sequentially in one-pot systems.
